# Transactions of the Chicago Gynæcological Society

**Published:** 1878-12

**Authors:** 


### Society Reports.

### TRANSACTIONS OF THE CHICAGO GYNÆCOLOGICAL SOCIETY.

*First regular meeting, October 25th,* 1878.

DeLaskie Miller, M. D., in the chair.

Dr. W. H. Byford read a paper on Dermoid Tumors of the Ovary,* of which the following is an abstract:

The paper included the histories of four cases, under the reader's observation, which were unlike in certain particulars. In Case I, an eighteen-year-old girl was tapped, for abdominal distension, in October, 1875; she had noticed her size increasing since the spring of 1874. Ten litres of a clear, slightly-bluish, somewhat tenacious fluid were drawn, which contained the ovarian cell. Ovariotomy was performed Jan. 4, the tumor having rapidly refilled in the meantime. Only a clear serum flowed through a Wells' trochar, but upon being opened, some 250 grams of sebaceous fat was found in the sac. The interior of the cyst was smooth, except at one point where an area as large as one's palm was depressed 2½ ctm. below the neighboring surface, and covered with a crop of brown hair about 2½ ctm. in length.

Case II was that of a woman forty-three years old, mother of one child aged eighteen years. Ovariotomy was done June 28, 1876, ten months after she began to notice an increase in her size. She was as large as a woman at term. The wall of the cyst was thin, except at one point where it contained a thick layer of adipose tissue, and was there 12 m. m. in thickness.

---

*The reader stated that he had presented the paper at the last meeting of the American Gynæcological Society.

Upon this area grew an abundant quantity of blonde hair, which was matted together in a mass as large as an orange. The hairs were from 16 to 40 ctm. long when straightened. The fluid was a thin serum, in which floated 320 to 380 grams of sebaceous fat, in considerable masses.

Case III was that of a woman thirty-three years old, mother of four children, the youngest aged three years. Ovariotomy was done April 7, 1875, about nine months after the abdomen began to enlarge. The wall of the cyst in three-fourths of its extent was thin; in the remaining portion, including that situated over the pedicle, the wall was made up of dense areolar tissue, which contained many cylindrical pieces of bone, varying in length from 2 to 5 ctm, and from 3 to 5 m. m. in thickness. They were loosely imbedded in the tissue. This loose areolar tissue had a tegumentary covering, to which more than a hundred imperfect incisor teeth were so slightly attached that they could be scraped off with the finger nail. Short, blonde hairs also sprung from this covering, interspersed with the teeth. The cyst contained some 10 litres of fluid, with several grams of yellow, sebaceous, fatty matter.

Case IV, thirty-five years old, mother of four children, the youngest twenty months old. Ovariotomy was performed June 18, 1878. The growth was first noticed nine years before. Upon tapping the cyst, at the operation, nothing but a sticky, greasy matter escaped. The cyst was divided by complete septa into three nearly equal compartments. Each space contained a ball of hair as large as a lemon, in which the fatty matter was intermixed. One ball was of blonde, another of red, and the third of gray hair. The hair on the woman's scalp was dark brown. Some of the hairs were 50 ctm. long, and all sprang from a tegumentary membrane resembling the scalp. By the side of the dermic patch, was a layer of loose areolar tissue, 4 ctm. thick, which contained pieces of bone in a variety of shapes and sizes. Upon this latter area of tissue, in each division of the cyst, was a half arch of teeth, resembling one half of the upper jaw. In one instance, the teeth projected above the surface, but were covered, in the other two spaces, by a thin covering, so soft that it could be removed with the fingers. Each arch contained an incisor and three molars ; two of the latter were shown.

In all these instances there was an absence of adhesions of the tumor with the abdominal walls, and of all other serious complitions. The patients all recovered.

The reader defined a dermoid tumor to be a cyst whose lining was wholly or in part a tegumentary membrane. It may be found in any region of the body. The usual contents of these cysts are the products of the skin : hair, sebaceous fat, and perspiratory fluid. Teeth, bones, muscular, nervous and even brain tissue are also found, but with less frequency. The latter are usually covered by the dermic tissue, or are found beneath that part of the cyst wall not lined by the dermic membrane.

Concerning the origin of these tumors, the reader said that the theories which had been advanced to explain them, in some degree represented the state of physiological science of the period. The explanation which to-day seemed, at least, physiologically plausible, had reference to the early days of the embryo, when, in some manner, an indentation of the external blastodermic layer becomes included in the anomalous situation, and there completes its development with the formation of hair, etc. The reason why these tumors are of greater size in the ovary than elsewhere, may be found in the nervous and vascular activity of which the organ is the seat ; and this explains why a dermoid cyst of the ovary is often undiscovered before puberty, when the organ is comparatively inactive.

### Discussion.

Dr. Jackson said he trusted that this society would never become one of mutual admiration. Its objects were of a much higher character—the principal one being the mutual benefit of its members. He mentioned this in order that he might not be misunderstood when he said that he felt himself fortunate in having the privilege of listening to the really excellent paper of Dr. Byford. He confessed that it had given him a clearer understanding of the subject of dermoid tumors than he had ever had before. The paper was very remarkable in many respects. Dr. Byford had been unusually fortunate in having the opportunity of seeing and treating so many as four cases of this really rare disease. Spencer Wells, in 500 cases of ovariotomy, had found that only nine of the tumors removed were of the dermoid variety ; Atlee, in 400 cases, found six ; and in a table given by Peaslee of 61 cases, only one was dermoid. Taking these as a basis for calculating, the percentage of dermoid tumors to other tumors of the ovary, was not more than 1½ or 2. As Dr. Byford's opportunities had been great, so also had he made the very best use of them. But the mysteries surrounding the subject had not by any means been cleared up. One of them related to the extraordinary number of teeth sometimes found in these tumors. Schnabel, quoted by Wells, instances a girl aged 13, in whom was an ovarian cyst of large size, containing three pieces of bone and more than 100 teeth ; Paget also mentions a case in which the cyst contained over 300 teeth. How can this be accounted for ? If the teeth are produced from bone in the ordinary manner, how is it that these pathological specimens in which are found only small pieces of bone and patches of tegumentary tissue, can produce so many more teeth—some well developed—than are found normally ? And what is the explanation of the fact that the hairs found in dermoid ovarian cysts are so frequently found in the form of masses or balls ? He would like to inquire whether, when on tapping a cyst we found the whole or a portion of the fluid removed become of the consistence of butter, we could be safe in assuming that the tumor was of the dermoid character.

Dr. Nelson : The theory proposed to account for the formation of dermoid tumors certainly seems a reasonable one, if we remember the number of organs or parts of organs normally formed by involution of the blastodermic cells, as, for example, the crystalline lens of the eye, from the epithelial cells of the skin ; the enamel of the teeth, from the same cells which form the epithelium of the mucous membrane of the mouth ; the hairs and the glands of the skin from the same cells which form the epidermis ; and especially the Graafian follicles of the ovary from the same cells which cover the peritoneum. If we remember also the numerous grooves and folds, fissures and processes formed in the blastodermic membranes during the development of the face, we need not be surprised to find this region a frequent seat of dermoid cysts. And especially will the development of the Wolffian bodies and their successors, the ovaries and testes, account for the frequent location of these tumors in this region. The Wolffian body is developed from cells in the middle blastodermic layer, just beneath the external blastodermic layer, which finally forms the skin and its appendages. It then descends toward the peritoneal cavity, carrying with it the external blastodermic layer sufficiently to form a groove, and thus aid in forming the Wolffian ridge from which the lower limbs are to be finally developed. This possibly accounts for the finding of these tumors occasionally in the thigh, the involution having really occurred at the Wolffian body and been carried downward in the wonderful development of the lower extremities at this period, instead of following the Wolffian body to the region of the testis, ovary or kidney. So we may consider these dermoid tumors a kind of complement to that other freak—cleft palate and hare-lip—the one a deficiency of blastodermic development and the other an excess.

Dr. Merriman thought Dr. Byford's method of bringing out these theories was very interesting; so also was the fact to which he called attention, that "these theories represent with some degree of exactness the physiology of the times in which they originated." The *scientific theory* given, certainly best explains the development of these strange cysts; but many puzzling facts are left unaccounted for, even in this theory. For example, it would naturally be expected that if the blastoderm had a definite constructive duty to perform, and a part of the membrane should be shut off from work with the rest, then the remainder would do an incomplete work, and the fœtus would be deficient in some respects. Or, on the other hand, if so small an amount of the blastoderm is included in the cyst that its loss is not missed in the construction of the fœtus, why then is there found in these cysts such immense development of some tissue which is generally supposed to be produced in the human body *only within a certain definite limit.* For instance, he was present at one of Dr. Byford's operations—not mentioned in this paper—when he found nearly a hundred teeth, more or less perfect, imbedded in the walls of the cyst. Dr. Byford will remember the case. Why should that trifling indentation in the blastoderm produce larger quantities of this tissue than is produced by the whole blastoderm in the fully developed body. These things are an enigma.

Dr. Sawyer: I do not presume to be able to add to the stock of knowledge which has been laid before the society in the paper just read. I only desire to remark that the differentiation of a dermoid tumor from those tumors which are known as monstrosities by inclusion, cannot, in all instances, be established. Many instances of the latter forms of tumor have become historical: notably the case of the man in France, who bore a large tumor of the scrotum. Coming under the observation of Velpeau, this remarkable diagnostician exclaimed, in a manner characteristic of his race, "My friends, that tumor is the man's brother," which, in fact, it proved to be. A second instance is that of the Chinese, from the anterior surface of whose thorax a second child sprouted; at birth this was as large as the first. A cast of this monstrosity, which is preserved in the Warren museum, at Harvard, represents this second child as having leaped into the thorax of the first. A third remarkable instance occurred to Dr. Joseph Pancoast, of Philadelphia, who removed a cystic tumor from the cheek of a very young child. The cyst contained a monster which, at the time of its removal, was growing faster than the independent child.

Monstrosities of this kind are usually regarded as quite distinct from the tumors described in the paper. The presumed point of departure being, for the subject of a dermoid tumor, that some portion of the individual's own external blastoderm has descended into, and been included, and shut off in some one of the numerous fissures which are found in the early embryo; while in a monster by inclusion, the entire or a portion of a neighboring embryo has been in a similar manner imprisoned in an embryonic fissure.

It is a curious and, perhaps, significant fact, that in these imprisoned monsters there is a tendency to an exaggerated development of the hairy portions of the integument and of the teeth. The cyst in Pancoast's case also contained much fatty matter. These and other facts have led Dr. Atlee, in his work, to describe monsters by inclusion in the same chapter with dermoid tumors. For the same reasons, other observers have thought it proper to look upon all dermoid tumors as instances of monstrosity by inclusion.

Dr. Earle said in regard to the accepted theory of the formation of these tumors, that it was exceedingly difficult for him to understand one step in the process. If these growths are the result of a depression of the blastoderm, which is finally severed from its proper place of development, how and why is it that in nearly *every case* this depressed portion is from that part of the blastoderm upon which teeth and hair especially are developed? Or, if this depressed portion is not from the same part of the blastoderm, shall we conclude that any depressed part of this membrane which is cut off from its proper place of growth has a tendency to develop teeth and hair?

Dr. Miller: I have been much interested in the paper read this evening. The history of the cases is instructive, and the deductions are ingenious. There are some points, however, relating to the genesis of dermoid cysts, which still remain occult. Would it not be pertinent to raise the query, whether dermoid tumors originate only by the inclusion of a portion of the blastoderm proper to the embryo? This theory may be accepted as a reasonable explanation of the origin of some.

There appears to me good reasons for believing that an embryo in the early stages of development may include another ovum, the development of which continues only for a limited period, and it may thus furnish the characteristic contents of a dermoid tumor. In the retrograde metamorphosis that ensues, it would be natural for the softer tissues to yield first to the changes, and the more resisting structures, such as teeth, bone, hair, nerve, etc., to remain. It is no disparagement to confess that much of our knowledge upon this subject is hypothetical.

Dr. Byford, in rising to close the conversation on his paper, said that he felt highly complimented at the manner it was received, and felt justified in reciprocating the encomium conferred upon it by expressing his satisfaction at the intelligence with which the members had criticised this abstruse subject. He acknowledged, in response to Dr. Jackson's remarks, that although the theory he had adopted was the most satisfactory in the present light we had, many of the minor points connected with the structure of these tumors could not be explained. Why there was so great a redundancy of teeth for so small a space and matrix for development, he could not say. He thought, however, the matted condition of the hair could be easily explained. The hair is matted, not rolled together, and each hair may be straightened out by simply taking hold and drawing it up. When we remember that while the hair is growing the fat is being secreted and entangling it, and thus preventing it from assuming any other than a matted condition, the explanation becomes evident. He would not hesitate to pronounce a tumor dermoid from which a fat flowed, at first fluid, and afterwards became solidified. At least he had always found this coincidence to be true evidence in favor of that sort of tumor.

Dr. Nelson has elaborated the theory from the standpoint occupied by the physiologist, in a manner that gave him great satisfaction. The remarks in this connection, he thought, must be accepted by the society as reasonable, and in accordance with the teachings of that branch of our science to which he has devoted himself.

In reference to what Dr. Sawyer said of monstrosities by inclusion, Dr. Byford believed that there might in certain cases be great difficulty in distinguishing between dermoid tumors and these engulfed monsters, during the life of the patient, and before they were removed, especially in the cavities of the body, or even when situated externally. He thought, however, that after the tumor had been removed, either during the life of the patient or *post mortem*, a careful inspection of its contents would remove all doubt as to its nature. In monstrosities by inclusion, the bones of the skeleton were sufficiently developed in shape and placed in their proper relation to admit of no doubt of their identity. The best instance of this description of heterotopy he remembered to have seen is to be found in Dr. Atlee's work on "Diagnosis of Ovarian Tumors." The dissection was made by Dr. Grant, and represented a fœtus so correctly that it would readily be recognized. In dermoid tumors, however, none of the bones are developed into unmistakable examples of femurs, humerus, maxilla or other bones in the skeleton. No development from the middle or inner layer of the blastoderm was complete, while some of the products of the external, or dermoid lamina were sometimes anatomically perfect, as the skin, hair and teeth.

It would not be strange to find, and probably if the possibility was borne in mind in making the investigation there would generally be found a continuation of the two, as the blastoderm of the including individual would be depressed at the point where the included ovum had been engulfed. There might, therefore, be a redundancy of hair, teeth and sebaceous fat produced by the dermic tissue, lining the cavity which accommodates the included fœtus.

In conclusion, the Doctor thanked the society for the interest manifested in his paper.

